# Supplementary material for: Tetraploidization Increases the Motility and Invasiveness of Cancer Cells
Source: Int J Mol Sci. 2023 Sep 10;24(18):13926. doi: 10.3390/ijms241813926 (PMC10531202; doi:10.3390/ijms241813926)
Supplement: Supplementary file 1 [file ijms-24-13926-s001.zip › ijms-2561374-supplementary.pdf]

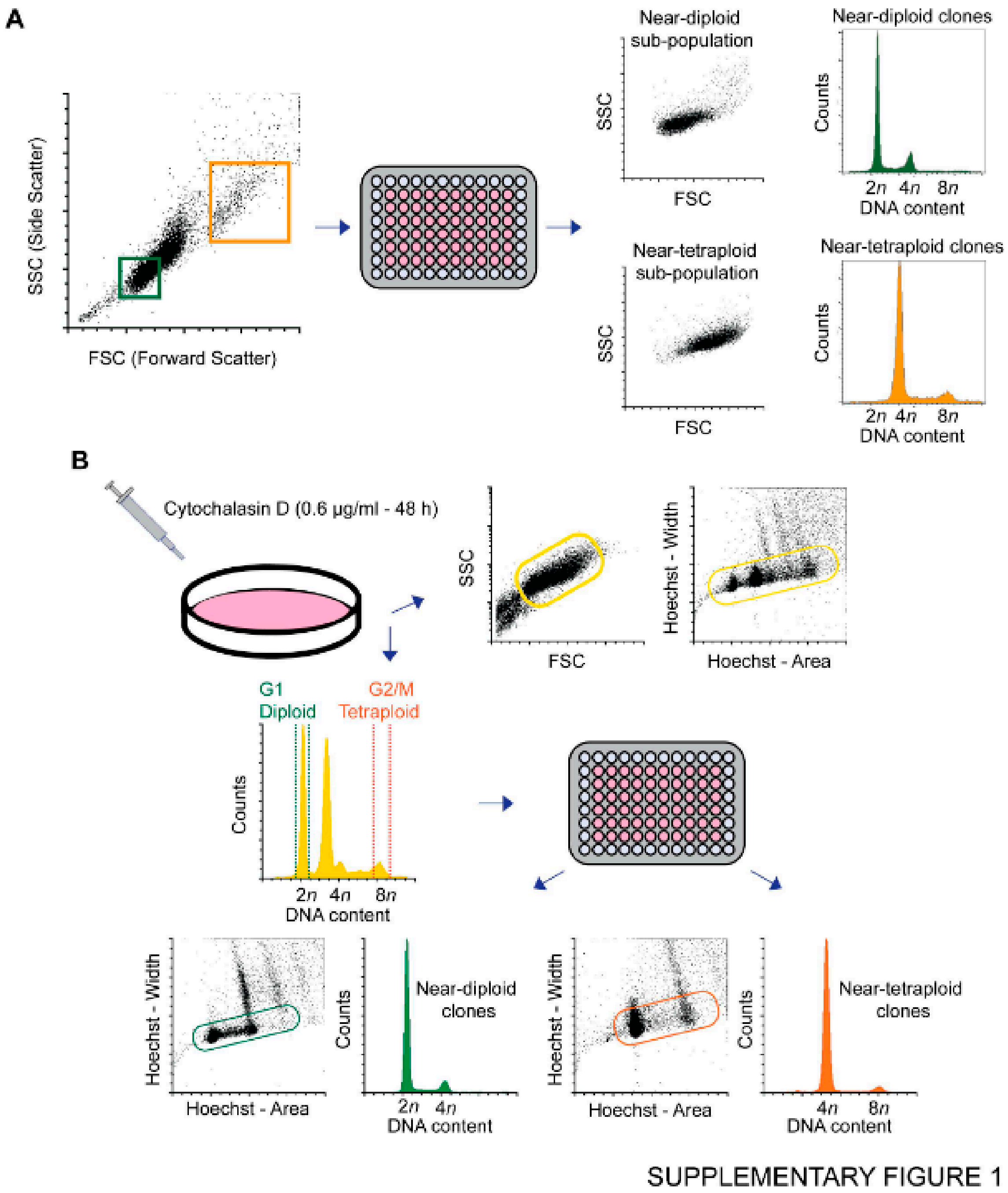

Figure S1. Generation and separation of diploid and tetraploid clones by Flow Cytometry. **A.** Separation of diploid and tetraploid cells based on size and granularity parameter using the normal light scattering parameters Forward Scatter (FSC) *vs* Side Scatter (SSC) gating. Individual clones were sorted and cultured in 96-well plates. Left panels show cycle profiles of the obtained sub-clones from small *vs* big cells. **B.** Separation of diploid and tetraploid cells based on cell cycle. Cells were treated with Cytochalasin D for 48 h and were subsequently stained with Hoechst 33342 for immediate FACS analysis. Pure tetraploid clones were sorted based on the tetraploid G2/M population (8n on the cycle axis) while pure diploid clones were sorted based on the diploid G1 population (2n on the cycle axis). Clones were cultured in 96 plate and colonies were transferred to 10 mL dish. Upper panel shows cycle profiles of the Cytochalasin D treated cells and mixed population of diploid and tetraploid cells, while lower panel shows pure diploid vs tetraploid sub-clones.

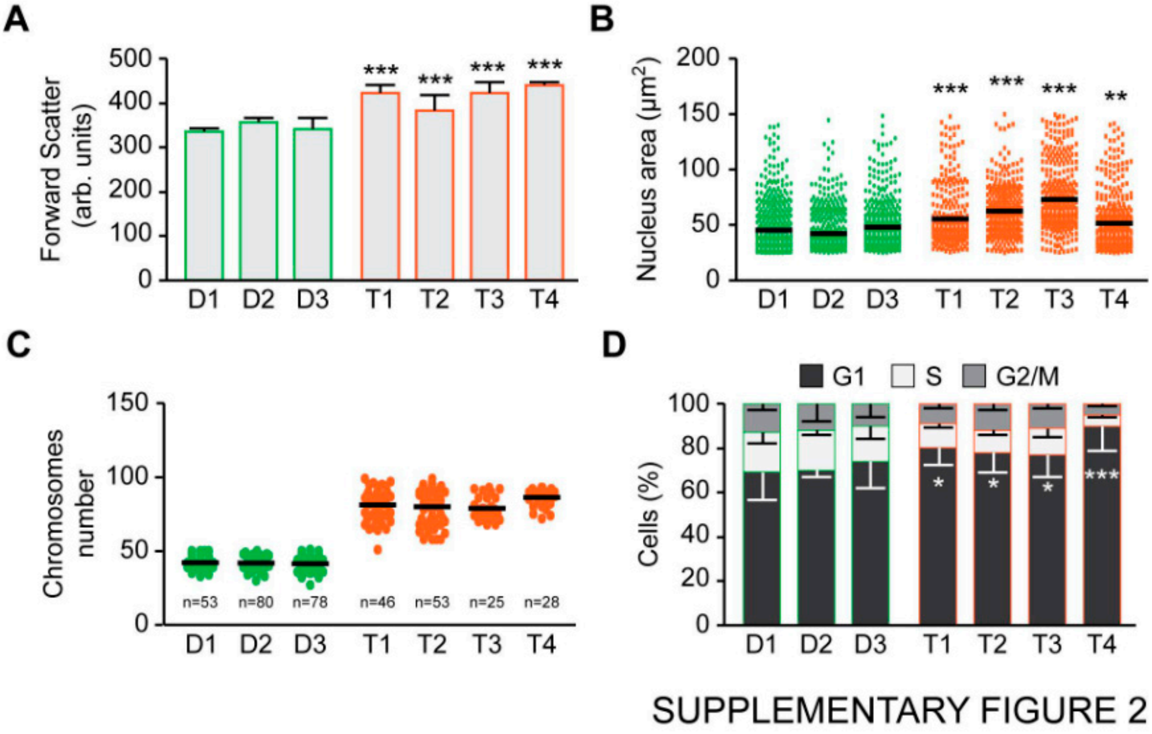

Figure S2. Sarcoma diploid vs tetraploid clones characterization. A. Cell size comparison between MFH152 diploid and tetraploid clones (labeled in green and orange respectively) using flow cytometry and light scattering parameters. B. Nucleus area analysis of MFH152 diploid and tetraploid clones. C. Chromosome number counts. Metaphase spread of diploid and tetraploid MFH152 clones was performed and quantitative data are displayed. D. Cell cycle analysis. Cell cycle distribution was assessed by flow cytometry. Diploid and Tetraploid MFH152 clones were collected and stained with propidium iodide. Quantitative data are reported. D refers for Diploid and T for Tetraploid clone, respectively. Data are reported as SEM; n = 5. * ($p < 0.05$), ** ($p < 0.01$) and *** ($p < 0.001$) indicate significant difference between every Tetraploid clone compared to Diploid clone D1 (using ANOVA test).
